# *In Vitro* and *In Vivo* Anticancer Effects of Sterol Fraction from Red Algae *Porphyra dentata*

**DOI:** 10.1155/2013/493869

**Published:** 2013-08-26

**Authors:** Katarzyna Kazłowska, Hong-Ting Victor Lin, Shun-Hsien Chang, Guo-Jane Tsai

**Affiliations:** ^1^Department of Food Science, National Taiwan Ocean University, 2 Pei-Ning Road, Keelung 20224, Taiwan; ^2^Center for Marine Bioenvironment and Biotechnology, National Taiwan Ocean University, 2 Pei-Ning Road, Keelung 20224, Taiwan

## Abstract

*Porphyra dentata*, an edible red macroalgae, is used as a folk medicine in Asia. This study evaluated *in vitro* and *in vivo* the protective effect of a sterol fraction from *P. dentata* against breast cancer linked to tumor-induced myeloid derived-suppressor cells (MDSCs). A sterol fraction containing cholesterol, **β**-sitosterol, and campesterol was prepared by solvent fractionation of methanol extract of *P. dentata* *in silica* gel column chromatography. This sterol fraction *in vitro* significantly inhibited cell growth and induced apoptosis in 4T1 cancer cells. Intraperitoneal injection of this sterol fraction at 10 and 25 mg/kg body weight into 4T1 cell-implanted tumor BALB/c mice significantly inhibited the growth of tumor nodules and increased the survival rate of mice. This sterol fraction significantly decreased the reactive oxygen species (ROS) and arginase activity of MDSCs in tumor-bearing mice. Therefore, the sterol fraction from *P. dentata* showed potential for protecting an organism from 4T1 cell-based tumor genesis.

## 1. Introduction

Breast cancers constitute one of the most serious problems in oncology, and in many countries breast cancer is a leading cause of death among women [1]. As the breast cancer phenotype progresses, survival factors that inhibit apoptotic cell death are expressed [2, 3]. In addition, the pathophysiology of breast cancer has linked it to tumor-induced myeloid derived-suppressor cells (MDSCs), and MDSCs pose a serious barrier to effective T cell immunotherapy against cancer [4]. 

Tumor-induced immune suppression is widespread among patients and experimental animals that display malignant tumors [5]. Multiple mechanisms have been suggested as the possible cause of tumor-induced immune suppression, with MDSCs (previously called immature myeloid cells) [6] being a major contributor [3]. In mice, MDSCs are characterized as Gr-1^+^CD11b^+^ cell phenotype and are thus also called Gr-1^+^CD11b^+^ cells [7]. Myeloid lineage differentiation antigen Gr-1 (also known as Ly6G) is expressed on myeloid precursor cells and granulocytes and transiently on monocytes [6]. Tumor growth is associated with systemic expansion of Gr-1^+^ myeloid cells with the CD11b antigen. The CD11b antigen is also known as *α*M-integrin, and is a marker for myeloid cells of the macrophage lineage [8]. The quantity of these suppressor cells is positively correlated to malignancy, which suggests that MDSCs play a role in tumor invasion and metastasis [7]. MDSCs in the bone marrow and spleen are significantly overproduced in tumor-bearing mice [7]. The expansion of MDSCs is promoted by expansion factors produced by tumor cells, through stimulation of myelopoiesis and the inhibition of differentiation of mature myeloid cells [3].

Some studies have suggested that MDSCs are potent inhibitors of antitumor immunity in secondary lymphoid organs and may facilitate tumor progression by inactivating antigen-specific T cells [6, 9]. MDSCs in tumor-bearing hosts may generate reactive oxygen species (ROS) to suppress antigen-specific cell responses [6, 10]. ROS inhibition can completely abrogate the suppressive effect of MDSCs [6]. MDSC suppression has also been implicated in reduced bioavailability of L-arginine [9, 11–13]. This effect is the result of enhanced activity of arginase, which hydrolyzes L-arginine to L-ornithine and urea. The reduction of L-arginine is thought to affect multiple key biological processes in T cells, including proliferation, expression of the T cell receptor complex, and the development of memory [9]. Zea et al. [9] showed that cytokines of interleukin 4 (IL-4) and IL-13 upregulate arginase activity of MDSCs, thereby increasing the suppressive function of MDSCs. *In vivo* studies have shown that blocking arginase eliminated the suppressor function of MDSCs and induced an antitumor effect of T cells [9, 13].

*In vitro*, 4T1 cells grow as adherent epithelial type and are characterized as murine mammary carcinoma cells [14]. The 4T1 cell line is an estrogen-nonresponsive [15] and transplantable tumor cell line that is tumorigenic in NOD/SCID and BALB/c mice. It multiplies rapidly, resulting in highly metastatic tumors in the lung, liver, lymph nodes, and brain while the primary tumor is growing *in situ* [4]. This tumor closely imitates human breast cancer and provides an animal model for Stage IV human breast cancer [2]. 

Algal remedies from the *Porphyra* species, commonly known as red seaweeds, are emerging as popular agents for cancer-related therapy [16, 17]. Various sterol components have been identified in the *Porphyra* species, including campesterol, cholesterol, 22-dehydrocholesterol, desmosterol, fucosterol, *β*-sitosterol, and stigmasterol [18–20]. Several of these components have been shown to exhibit antitumor activity [21]. In eastern Asian countries, *Porphyra dentata* (Bangiaceae) has been used for centuries in the preparation of folk medicine [22, 23]. This study investigated the anti-breast-cancer effects of *P. dentata *using the 4T1 breast cell line *in vitro* and 4T1 implanted mice *in vivo*. We analyzed the presence of effective compositions of sterols and attempted to explain the underlying mechanism related to MDSC to account for these anticancer effects. 

## 2. Materials and Methods

### 2.1. Plant Materials and Chemicals

Marine red alga, *P. dentata*, was collected in Taiwan and authenticated as described previously [22]. Unless otherwise stated, chemicals and reagents were purchased from Sigma Aldrich (St. Louis, MO, USA). These included aprotinin, L-arginine, campesterol, cholesterol, desmosterol, dichlorodihydrofluorescein diacetate (DCFDA), dimethyl sulfoxide (DMSO), 3-(4,5-dimethylthiazol-2-yl)-2,5-diphenyltetrazolium bromide (MTT), EDTA (ethylenediaminetetraacetate), *α*-isonitrosopropiophenone, N-(1-naphthyl) ethylenediamine, leupeptin, phenyl methyl sulfonyl fluoride, *β*-sitosterol, stigmasterol, sulfanilamide, taxol, tris, triton X-100, and urea. We obtained catalase, arginase inhibitor N^W^-hydroxy-nor-L-arginine (nor-NOHA), and phorbol-12-myristate-13-actetate (PMA) from Calbiochem (Calbiochem, San Diego, CA). Fetal bovine serum (FBS), EDTAx4Na (tetrasodium EDTA), HEPES buffer, 2-*β*-mercaptoethanol, nonessential amino acids, penicillin/streptomycin, sodium pyruvate, Dulbecco's Modified Eagle's Medium (DMEM), PBS, and RPMI 1640 were purchased from Gibco (Rockville, MD, USA). From BD Biosciences (San Diego, CA, USA) we obtained a fluorescein isothiocyanate (FITC) Annexin V Apoptosis Detection Kit I, propidium iodide (PI), and the antibodies, namely, anti-mouse Gr1^+^-FITC (Clone: RB6-8C5; Catalog number: 553126) and anti-mouse CD11b^+^-PE (Clone: M1/70; Catalog number: 553311). A protein assay kit (Bradford reagent) was purchased from Bio-Rad Laboratories (Richmond, CA, USA). All solvents and chemicals used in this study were of HPLC grade.

### 2.2. Extraction and Fractionation of Plant

Crude extract of *P. dentata* soaked in methanol was prepared as described previously [22]. A freeze-dry powder of the methanolic crude extract (18 g) was fractionated on a chromatographic column filled with silica gel particles. The first fractionation was eluted with 1 L of hexane (fraction 1; F1), followed by 2.5 L of dichloromethane (fraction 2; F2), 3 L of ethyl acetate (fraction 3; F3), 4 L of acetone (fraction 4; F4), and 4 L of methanol (fraction 5; F5). The various obtained fractions were concentrated in a rotary evaporator to dryness and were then weighed and stored in a freezer (−80°C) until use. 

### 2.3. Cell Line Maintenance

The 4T1 breast cancer cell line was obtained from the American Type Culture Collection (ATCC) and was maintained using standard cell culture techniques. The cells were cultured in a “4T1 medium” [24] comprising DMEM supplemented with 0.2% (w/v) NaHCO_3_, 10% (v/v) FBS, and 1% (w/v) penicillin/streptomycin; the culture was maintained at 37°C, 95% humidity, and 5% CO_2_. The subcultures were performed every 3 days. The cells were detached using 0.05% (w/v) trypsin with 0.53 mM EDTAx4Na for 5 min at 37°C in a humidified incubator. The cell count for each subculture was determined using a hemacytometer (Marienfeld, Long Isand, NY, USA). 

### 2.4. Cytotoxic Effects of Samples on 4T1 Cells

The 4T1 cells (1 × 10^6^ cells/mL) were incubated with 4T1 medium containing DMSO (control), various concentrations of methanolic crude extract (100, 200, and 400 *μ*g/mL), or fractions of F1, F2, F3, F4, and F5 (25, 50, and 100 *μ*g/mL in each case) for 48 h. The cells were then subjected to MTT assays [22]. The relative cell viability (%) was defined as 100 multiplied by the ratio of the OD490 value of the cells treated with extract or fraction, over the OD490 value of the control cells. 

### 2.5. *In Vitro* Effect of Sample on Apoptosis and Necrosis of 4T1 Cells

The 4T1 cells (1 × 10^6^ cells/mL) were incubated in the 4T1 medium containing various concentrations of F2 fraction for 48 h. The cell undergoing apoptosis or necrosis was determined using FITC Annexin V Apoptosis Detection Kit I and PI exclusion in a FACS-based assay, according to the manufacturer's protocol (BD Biosciences, San Diego, CA, USA). Briefly, the reacted 4T1 cell suspension was mixed with 1 *μ*L of FITC Annexin V staining solution and left for 7 min. Thereafter, 1 *μ*L of PI solution was added and reacted for 15 s in the dark. Flow cytometric analysis was performed immediately. 

### 2.6. Mouse Maintenance

Thirty female BALB/c mice (5 wk old) were purchased from National Laboratory Animal Center (NLAC) in Taiwan. They were fed *ad libitum* for 1 wk and were then divided randomly into 5 groups of 6 animals each. The groups were as follows: a naive group of tumor-free mice, a control group of tumor-bearing mice that did not receive treatment, and 3 experimental groups of tumor-bearing mice that received the sample treatments at 3 dosage levels. For the survival rate analysis, another 30 female BALB/c mice (5 wk old) were divided in the same manner. The 6 mice in each group were housed in a cage at 25°C and were libitum fed and observed daily. Approval for this study was issued by the Institutional Animal Care Board of National Taiwan Ocean University, and the mice were handled and euthanized according to the board's guidelines.

### 2.7. 4T1 Breast Tumor Model

The experimental mice were injected with 100 *μ*L of syngeneic breast cancer 4T1 cell suspension (5 × 10^6^ cells/mL). The injection was delivered subcutaneously (s.c.) into the mammary fat pads of the mice. Three hours after the tumor engraftment, the experimental mice (3 groups) were intraperitoneally (i.p.) injected with 20 *μ*L of sample at dosages of 5, 10, or 25 mg/kg/day. The negative control group (naive mice) and the control group (tumor-bearing mice) were injected with DMSO (a carrier). The mice were injected every 3 d over 18 consecutive days. 

Tumor growth was assessed morphometrically, using a caliper, and tumor volumes were calculated from the formula *V* (mm^3^) = *L*_1_ (major axis) ×  *L*_2_^2^ (minor axis)/2 [25]. After the indicated time, the mice were sacrificed on frozen CO_2_. Gross pathology of animals was performed, abnormalities were noted, and images were obtained to document the results. Tumor tissues were isolated and weighed on a microbalance and then stored in cytotoxic media on ice until further *ex vivo* analysis. The cytotoxic media comprised 1 mM nonessential amino acids, 10 mM hepes buffer, 50 *μ*M 2-*β*-mercaptoethanol, 1% (v/v) sodium pyruvate, 2.0 g/L NaHCO_3,_ and 100 U/mL penicillin/streptomycin in RPMI 1640. 

### 2.8. Splenocyte Isolation

The suspensions of splenic cells were prepared according to the method of Nelson et al. [26]. The cell suspensions were washed once with media and the pellet was resuspended in erythrolysis buffer (7.47 g/L NH_4_Cl, 2.29 g/L KHCO_3_, 0.22 *μ*m filtrated, pH 7.2). Erythrolysis was performed at 37°C for 10 min in a water bath. Thereafter, the cells were washed twice with phosphate buffer saline (PBS) and filtered through a cell strainer (40 *μ*m, BD) to remove conglomerated or dead cell debris.

### 2.9. FACS Analysis of MDSC Surface Proteins in Splenocytes

Splenocytesat 1 × 10^6^ cells/well in a 96-well V bottom plate (Hartenstein) were spun down. Cells were resuspended in 50 *μ*L FACS buffer (1% v/v FBS, 0.05% w/v NaN_3_ in PBS) containing the selected antibody cocktail (fluorescence-conjugated antibodies Gr-1^+^-FITC and CD11b^+^-PE) and were then incubated at 4°C in the dark for 30 min. After staining, the cells were washed twice with FACS buffer and were resuspended in 500 *μ*L FACS buffer. A single stain was performed for each antibody present in the staining cocktail. After staining, the cells were analyzed using a BD FACSCanto II flow cytometer with FACSDiva software (BD Biosciences, San Diego, CA, USA). The instrument was thresholded on a forward angle light scatter (FS), and signals from FS and orthogonal light scatter (SS) were collected using a linear scale. Two colors of fluorescence were collected using logarithmic amplification, with the optical filters set at approximately 525 nm (on FL1 channel for FITC fluorochrome detection) and approximately 575 nm (on FL2 channel for PE fluorochrome detection).

### 2.10. Single-Cell Sorting of MDSC Fraction

Splenocytes were stained with fluorescence-labeled antibodies and sorted with a BD FACSAria II flow cytometer using FACSDiva software (BD Biosciences, San Diego, CA, USA). The MDSCs were freshly collected for assays of ROS production and arginase activity.

### 2.11. ROS Detection Assay

DCFDA, a cell-permanent dye sensitive to oxidation by ROS, was used to determine oxidative stress in the MDSCs isolated from the spleens of BALB/c mice [6]. We labeled 100 *μ*L of MDSCs (2 × 10^6^ cells/mL) in PBS using 1 *μ*L DCFDA (2 *μ*M) for 15 min at 37°C; thereafter, the cells were washed twice with PBS. The oxidized DCFDA-labeled cells were quantified in a green channel (FL1) of fluorescence by flow cytometry.

### 2.12. Arginase Activity Assay

Arginase activity was measured using the method of Chang et al. [27] with some modifications. To prepare cell lysates for arginase activity assay, the sorted MDSCs were rinsed twice with ice-cold Dulbecco PBS and then suspended in 300 *μ*L of lysis buffer containing 50 mM Tris-HCl (pH 7.5), 0.1 mM EDTA, 1 mg/mL leupeptin, 1 mg/mL aprotinin, 0.1 mM phenylmethylsulfonyl fluoride, and 0.4% Triton X-100. Finally, cells were lysed for 10 min in the lysis buffer and the arginase activity in the cell lysate was measured. In brief, cell lysate (5 *μ*g/50 *μ*L ddH_2_O) was added to 50 *μ*L of Tris-HCl (50 mM; pH 7.5) containing 10 mM MnCl_2_. The arginase in MDSCs was then activated by heating the mixture at 55°C for 10 min. The hydrolysis reaction of L-arginine by arginase was performed by coincubating the activated arginase with 50 *μ*L of L-arginine (0.5 M, pH 9.7) at 37°C for 1 h. Thereafter, 400 *μ*L of the acid solution (95% H_2_SO_4_ : 85% H_3_PO_4_ : H_2_O = 1 : 3 : 7) was added to stop the reaction. For colorimetric determination of urea, 25 *μ*L *α*-isonitrosopropiophenone (9% in absolute ethanol) was added. The mixture was heated at 100°C for 45 min and quickly cooled to 25°C. After being kept in the dark at 25°C for 10 min, the absorbance at 550 nm was determined using a microplate reader (lQuantTM, BIO-TEK Instrument Inc., Winooski, VT). The amount of urea produced was calculated from the standard curve and prepared as described but using urea (mg/mL) instead of cell lysate for the arginase activity assay. 

### 2.13. Identification of Sterols Using a High-Performance Liquid Chromatography Evaporative Light Scattering Detector (HPLC-ELSD)

The HPLC-ELSD system consisted of a pump PU-1580 obtained from Jasco (Tokyo, Japan), a Mightysil RP-18 (H) column (250 × 4.5 mm) preceded by an Asahipak GS-2G 7B guard column (50 × 7.6 mm) (Shodex, Showa Denko, Tokyo, Japan), and an ELSD ZAM 3000 (Schambeck SFD GmbH, Bad Honnef, Germany). The temperatures for the column oven and detector nebulizer were 40°C and 80°C, respectively. Samples were injected through a CO-150 sampler with a 20 *μ*L sample loop (Rheodyne, Cotati, CA, USA). The mobile phase was a mixture of methanol and deionized water at a ratio of 95 : 5 with a flow-rate of 1 mL/min. Commercially supplied campesterol, desmosterol, *β*-sitosterol, and cholesterol were used as calibration standards for this system. Chrome Manager 5.8 software from Analytical Based Development Center (Taichung, Taiwan) was used for online data monitoring and analysis.

### 2.14. Statistical Analysis

Survival curves were generated using Sigma-Plot software; the Kaplan-Meier method [28] and the logrank test [29] were used to compare mean survival. All other analyses were performed using Student's *t*-test and the results were expressed as means ± standard deviation (SD). Differences were considered significant at *P* < 0.05.

## 3. Results

### 3.1. Effects of Sterol Fraction of *P. dentata* on Proliferation and Apoptosis in 4T1 Cells

**Figure 1:**
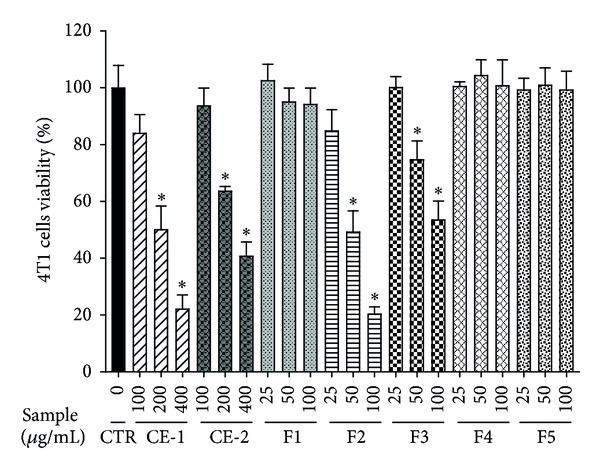
Effect of *P. dentata *methanolic crude extract (CE-1) and dichloromethane (DCM) crude extract (CE-2) as well as F1 (hexane), F2 (DCM), F3 (ethyl acetate), F4 (acetone), and F5 (methanol) fractions of CE-1 on the viability of tumor 4T1 cells. The 4T1 cells were incubated with DMEM containing DMSO (control), CE-1, CE-2, or indicated fractions for 48 h at indicated concentrations. The cells were subjected to MTT assays. The relative cell viability (%) was defined as 100 multiplied by the ratio of the OD490 value of the cells treated with sample over the OD490 value of the control cells. Data are expressed as mean ± S.D. (*n* = 3).

The cytotoxic effects of the methanol crude extract (CE-1) of* P. dentata* and various solvent fractions (F1–F5) of CE-1 on 4T1cells were investigated. As shown in Figure 1, cell viability decreased in a dose-dependent manner in samples of CE-1, F2, and F3. Cell viability was significantly decreased after treatment with CE-1 (200 or 400 *μ*g/mL), F2 (50 or 100 *μ*g/mL), or F3 (50 or 100 *μ*g/mL). Samples of F1, F4, and F5 did not affect cell viability. Furthermore, F2 was found to have the highest cytotoxic effect on 4T1 cell proliferation. Therefore, F2 was selected for testing in the following experiments.

**Figure 2:**
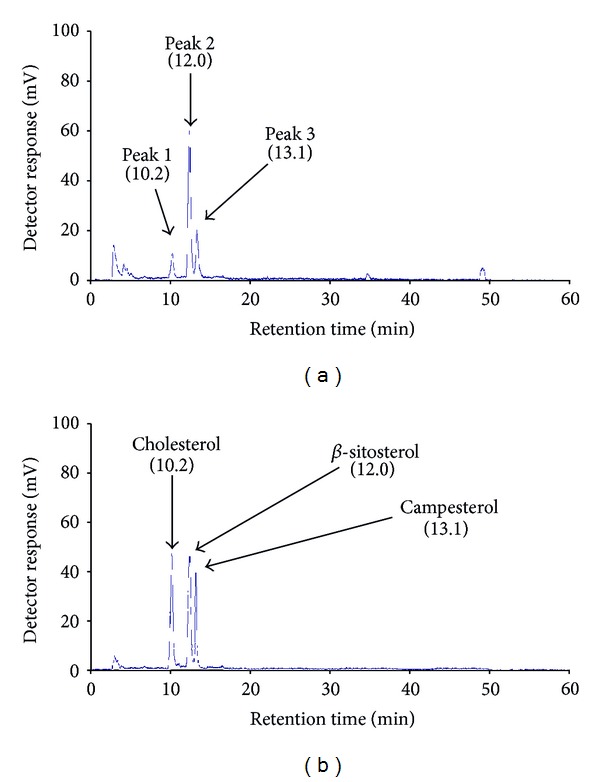
HPLC-ELSD chromatograms of *Porphyra dentata* F2 fraction (a) and a mixture of standards (cholesterol, *β*-sitosterol, and campesterol) (b). The retention time (*T*_*R*_) of each major peak (Peak 1, Peak 2, and Peak 3) present in F2 fraction and each standard is shown in parentheses and on a time scale. Conditions for HPLC-ELSD: column, Mightysil RP-18 (H) column (250 × 4.5 mm); column temperature, 40°C; mobile phase, 95% methanol in water; flow rate: 1 mL/min; ELSD detector nebulizer temperature, 80°C.

The sterol components in F2 were determined by HPLC-ELSD after comparing the retention time (*T*_*R*_) of individual peaks with the *T*_*R*_ library of sterol standards for cholesterol, *β*-sitosterol, and campesterol. The data were analyzed under the same HPLC conditions (Figure 2). The three major peaks in F2 had retention times of 10.2 min (Peak 1), 12.0 min (Peak 2), and 13.1 min (Peak 3) (Figure 2(a)), which were equivalent to those of cholesterol (*T*_*R*_ = 10.2 min), *β*-sitosterol (*T*_*R*_ = 12.0 min), and campesterol (*T*_*R*_ = 13.1 min), respectively (Figure 2(b)). The relative weight percentages for cholesterol, *β*-sitosterol, and campesterol in F2 were 15%, 55%, and 30%, respectively.

**Figure 3:**
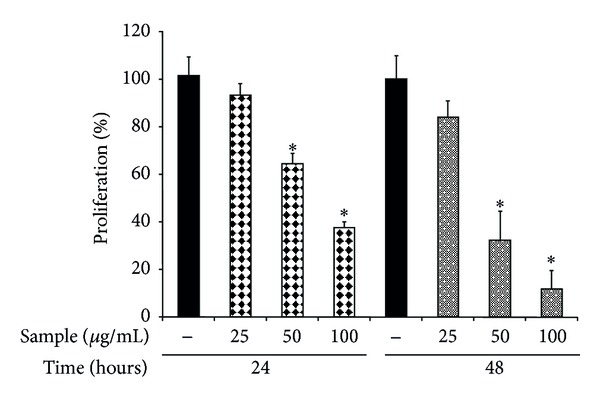
Effect of *Porphyra dentata* F2 fraction on viability of 4T1 cells. The 4T1 cells were incubated in DMEM culture containing 10% FBS and sample at indicated concentrations for 24 h and 48 h. Thereafter, the cells were subjected to MTT assay. The proliferation percentage (%) was defined as 100 multiplied by the ratio of the OD_490_ value of the cells treated with sample over the OD_490_ value of the control cells. Data are expressed as mean ± S.D. (*n* = 3).

The dose effects of F2 on 4T1 cell proliferation and apoptosis were further investigated. As shown in Figure 3, the proliferation of 4T1 cells was inhibited by the sample (F2) in dose- and time-dependent manner. The proliferation percentage of 4T1 cells was significantly decreased by a dose of 50 *μ*g/mL of F2, compared with the control cells. The proliferation percentage in treated 4T1 cells was 63.3% compared with the control cells at 24 h; by 48 h it had reduced further to 31.1%. Based on the data shown in Figure 3, the sample concentrations required to achieve 50% inhibition of cell proliferation at 24 h and 48 h were calculated as 76.2 and 48.3 *μ*g/mL, respectively. 

**Figure 4:**
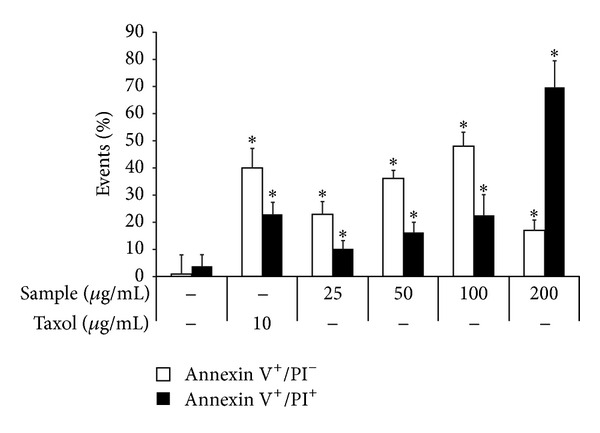
Simultaneous *in vitro* detection of apoptosis (annexin V staining) and necrosis (PI staining) of 4T1 cells treated with *P. dentata* F2 fraction. The 4T1 cells were incubated in DMEM culture containing 10% FBS and F2 sample at indicated concentrations for 48 h. Taxol (10 *μ*g/mL) was used as a positive control. The relative cell population (%) undergoing a solely apoptotic (annexin V positive) pathway is shown by an open bar square; cells undergoing necrotic-apoptotic pathway (annexin V and PI double positive) are shown by a closed black bar square. Data are expressed as mean ± S.D. (*n* = 3).

PI and annexin V dual staining enables the identification of live cells (using annexin V^−^ and PI^−^); early membrane damage (using annexin V^+^ and PI^−^); and apoptotic, necrotic, or necrotic membrane-disrupted cells (using annexin V^+^ and PI^+^). To determine if the ability of the sample to inhibit proliferation was connected with apoptosis in 4T1 cells, dual staining of annexin V and PI for cells undergoing necrosis-apoptosis was performed at 48 h (Figure 4). Less than 5% of 4T1 cells in control were dual positive for annexin V and PI (Figure 4). In the presence of the sample at dosages of 25 to 100 *μ*g/mL, the percentage of apoptotic cells (white bar) and apoptotic-necrotic cells (black bar) gradually increased from 22% to 50% and from 10% to 20%, respectively. In the presence of 200 *μ*g/mL of the sample, the percentage of apoptotic-necrotic cells increased to 70%, and the apoptotic cell percentage decreased to 15%. The remaining 15% of events showed nonstained viable cells (Figure 4). In this assay, taxol, a clinical chemotherapy drug for breast and ovarian cancer, was used as a positive control. Administering taxol at a dosage of 10 *μ*g/mL induced cell apoptosis at a level of 40% (white bar) and apoptosis-necrosis at 22% (black bar) (Figure 4).

### 3.2. Antitumor Effects of Sterol Fraction of *P. dentata In Vivo*

**Figure 5:**
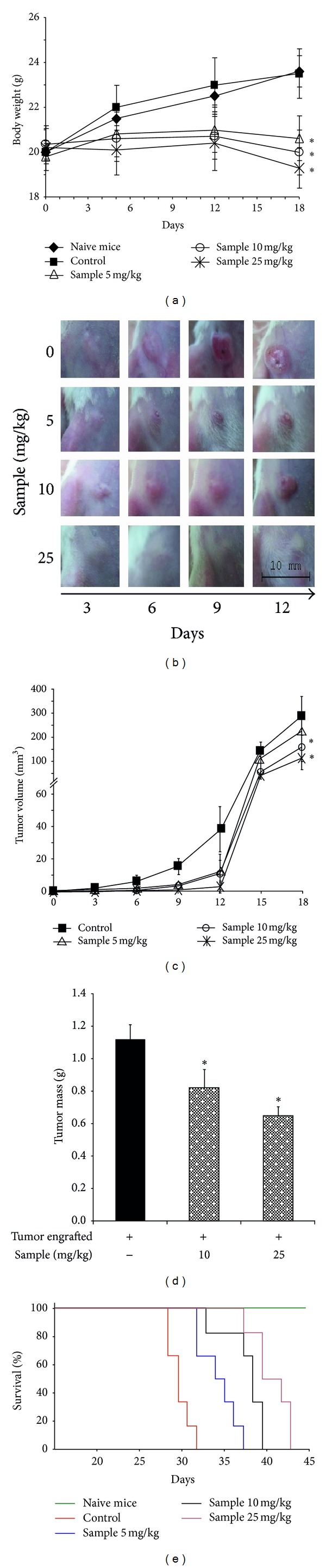
Inhibitory effect of *P. dentata* F2 fraction on 4T1 tumor-cell-engrafted mice. The 4T1 cells were subcutaneously engrafted (5 × 10^5^ cells suspended in 0.1 mL of PBS) in the nipples of BALB/c female mice. The following parameters were measured: (a) body weight, (b) photo for tumor growth status, (c) primary tumor volume, (d) primarytumor mass, and (e) survival rate. The data are representative of one experiment with 6 mice in each group. Naive mice and control mice were i.p. treated with DMSO only.

The 4T1 cell-implanted tumor mice were i.p. injected with sample every 3 d over 18 consecutive days. Changes in the body weights and tumor sizes of mice were monitored. After the animals were sacrificed, the primary tumor nodules were isolated and weighed. On day 18 of the experiment, the body weight of tumor-bearing mice in groups that were treated with the sample was significantly decreased, compared with the tumor-bearing control group, for all 3 dosage levels (Figure 5(a)). As shown in Figure 5(b), tumor nodules developed by day 3 in tumor-bearing control mice, whereas tumor nodules were barely detectable on days 3 and 6 in tumor-bearing mice that were treated with sample at a dose of 25 mg/kg. On day 18, the volume and mass of 4T1 tumors were significantly larger in the control group than in the tumor-bearing group treated with the sample at dosages of 10 and 25 mg/kg (*P* < 0.05) (Figures 5(c) and 5(d)). By contrast, on day 18 no significant difference was noted in tumor size between the control mice and mice treated with a low dose of the sample (5 mg/kg) (Figures 5(b) and 5(c)). Increased survival benefits were observed in the sample-treated groups (Figure 5(e)). All 6 mice in the tumor-bearing control group died within 33 days, whereas mice treated with sample at a dose of 25 mg/kg survived significantly longer, at 43 days (*P* = 0.039). Overall, these results showed that the sterol fraction of *P. dentata* effectively retarded tumorgenesis and increased mouse survival (Figure 5). 

### 3.3. Sterol Fraction of *P. dentata* Did Not Affect Gr-1^+^CD11b^+^ Myeloid Cell Expansion

**Figure 6:**
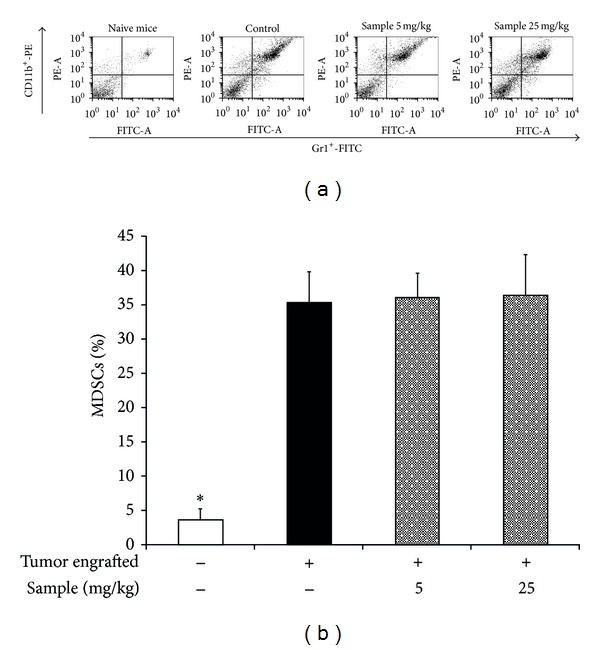
The induction of MDSCs in female BALB/c mice treated with or without *P. dentata* F2 fraction. The 4T1 cells were subcutaneously engrafted in the mice. A typical dot plot of splenic MDSCs level is shown in (a). The percentage of MDSCs detected in the spleen of mice bearing 4T1 tumors is shown in (b). The data are representative of one experiment, with 3 mice from each group.

The effect of the sterol fraction of *P. dentata* on the percentage of MDSC in splenocytes of tumor-bearing mice was investigated. The MDSCs were labeled with fluorescence-conjugated antibodies Gr-1^+^-FITC and CD11b^+^-PE and were then detected using flow cytometry. On Day 18, the percentage of splenic MDSCs in tumor-bearing control mice was markedly elevated by 36% compared with naive tumor-free mice (4%) (Figure 6). By contrast, mice treated with dosages of 5 or 25 mg/kg of sample did not show significant changes in the MDSC induction level, compared with tumor-bearing control mice (Figure 6). This result suggested that the sterol fraction of *P. dentata* did not inhibit MDSC induction pathways in BALB/c mice. 

### 3.4. Sterol Fraction of *P. dentata* Decreased ROS Levels in Purified MDSCs

**Figure 7:**
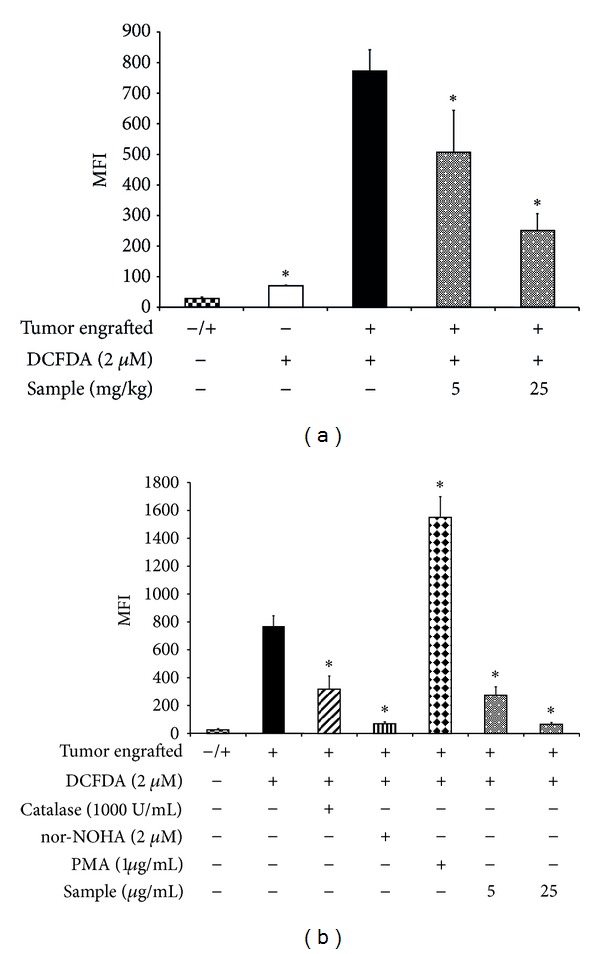
*In vivo* effect of *P. dentata* F2 fraction (a) and *in vitro* effect of F2 fraction and various agents (b) on ROS production by MDSCs. *In vivo*, tumor-bearing BALB/c mice were i.p. injected with F2 fraction at dosages of 5 and 25 mg/kg body weight. MDSCs were isolated from tumor-bearing control mice and F2-treated mice as described in Section 2 and their ROS productions were measured. *In vitro*, MDSCs isolated from the tumor-bearing control mice were incubated first with F2 fraction or the tested agent; thereafter, ROS production was measured. Where the stimulating agent PMA was used, MDSCs were incubated at 37°C for 5 min with 1 *μ*g/mL PMA and then washed with cold PBS. To block ROS production *in vitro*, MDSCs were incubated with catalase, arginase inhibitor (nor-NOHA), or F2 fraction at 37°C for 10 min. The oxidative-sensitive dye DCFDA was used to measure ROS production by the cells. MDSCs were incubated with 2 *μ*M DCFDA for 15 min at 37°C and then washed twice with PBS. The intensity of fluorescence was measured by flow cytometry. Data were obtained from one experiment, with 4 individual mice from each group. Measurements were obtained in duplicate and the mean fluorescence intensity (MFI) was calculated for MDSCs in each group, for both *in vivo* and *in vitro* treatments.

The ROS levels in MDSCs isolated from BALB/c mice using an oxidation-sensitive fluorescent dye, DCFDA, are shown in Figure 7. *In vivo*, MDSCs from tumor-bearing control mice exhibited 10 times more ROS-positive cells than MDSCs from tumor-free mice (Figure 7(a)). The mean fluorescence intensity (MFI) values represent the ROS content. These values were 65 ± 3 and 777 ± 68 for naive MDSCs and tumor-bearing control MDSCs, respectively. By contrast, the MFI values for MDSCs from tumor-bearing mice treated with the sample at dosages of 5 and 25 mg/kg were 621 ± 84 and 265 ± 50, respectively; both of which were significantly lower than the MFI values for MDSCs from the control group (Figure 7(a)). *In vitro*, the purified MDSCs from tumor-bearing control mice were treated with various stimuli, including catalase (a ROS-quenching agent), PMA (a ROS induction agent), nor-NOHA (an arginase inhibitor), and sample. As expected, the *in vitro* ROS level in untreated control MDSCs (MFI 776 ± 47) was similar to the *in vivo* MDSC level in the tumor-bearing control group (777 ± 68, Figure 7(a)). Treatment with catalase or nor-NOHA decreased the ROS level significantly to MFI 336 ± 67 and MFI 70 ± 3, respectively. By contrast, the ROS level of MDSCs treated with ROS-inducing PMA increased 2-fold (MFI 1556 ± 120). Similarly, the ROS levels of MDSCs treated *in vitro* with the sample at dosages of 5 and 25 *μ*g/mL were significantly decreased, with MFI values being 271 ± 66 and 72 ± 15, respectively (Figure 7(b)). Overall, the sterol fraction of *P. dentata* decreased ROS in a dose-dependent manner in MDSCs both *in vivo* and *in vitro* (Figure 7).

### 3.5. Sterol Fraction of *P. dentata* Inhibited Arginase Activity of MDSCs *In Vivo*

**Figure 8:**
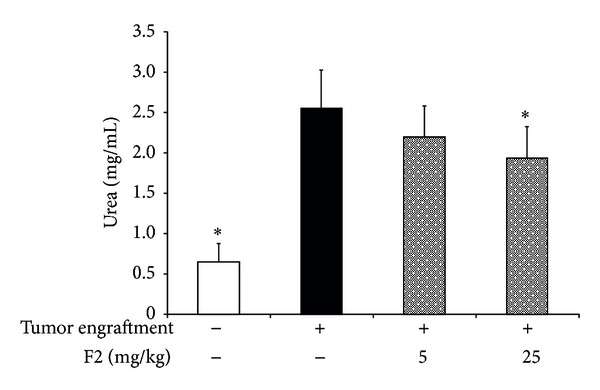
Arginase activity of MDSCs in naive mice, tumor-bearing control mice, and tumor-bearing mice treated with *P. dentata* F2 fraction. The splenic MDSCs were isolated from various groups of BALB/c mice and were lysed for 10 min in the lysis buffer and the arginase activity (shown as urea production) in the cell lysate was measured, as described in Section 2. Data were obtained from one experiment, with 3 mice from each group, and expressed as mean ± S.D.

Arginase activity is an important contributor to MDSC suppressive activity. Thus, the arginase activity in MDSCs from naive, control, and sample-treated mice was measured to determine whether the arginase activity of MDSCs was modulated *in vivo* by our sample. As shown in Figure 8, the arginase activity (expressed as urea level) in control MDSCs was 4-fold higher than that in naive MDSCs. No significant difference was found in arginase activity between the control MDSCs and cells treated with the sample (5 mg/kg). However, arginase activity (1.9 mg/mL of urea) in MDSCs from mice treated with sample (25 mg/kg) was significantly lower (*P* < 0.05) than that of the control (2.6 mg/mL of urea) (Figure 8). 

## 4. Discussion

*Porphyra dentata* is an edible seaweed that is widely cultivated in Taiwan. Our previous study examined the anti-inflammatory effect of *P. dentata *methanolic crude extract on iNOS-implicated diseases in lipopolysaccharide-challenged mouse macrophages [22]. The current study showed for the first time that dichloromethane solvent fraction (F2) of the crude extract of *P. dentata* displays anti-breast-cancer activities.

Phytochemical analysis showed that our biologically active sample was composed of cholesterol and phytosterols (*β*-sitosterol and campesterol) (Figure 2). Recent findings from a number of studies have shown that foods containing phytosterols, such as *β*-sitosterol either alone or in combination with campesterol, may protect animals from the risk of various tumors [21, 30–32]. Phytosterols exert anticancer actions by inhibition of carcinogen production, cancer cell proliferation, angiogenesis, invasion and metastasis, and induction of apoptosis of cancer cells [33]. Similarly, probably because of the presence of *β*-sitosterol and campesterol, our sample inhibited proliferation of breast cancer cells *in vitro* (Figure 3) and caused an apoptotic-necrotic effect in the cells (Figure 4). Furthermore, treatment with the sample effectively reduced the tumor size of 4T1 implanted cells and prolonged survival of the mice (Figure 5). 

Previous research has established that MDSCs play a key role in tumorgenesis [9, 11]. The expansion of MDSCs in bone marrow of tumor-bearing hosts is governed largely by factors produced by tumor cells, which results in an accumulation of MDSCs in the secondary lymphoid organs (spleen and lymph nodes) [3]. This process occurs through the stimulation of myelopoiesis and inhibition of the differentiation of mature myeloid cells [3, 34]. Similar expansion, although not inhibited by the sterol fraction of *P. dentata*, was observed in splenic MDSCs of tumor-bearing mice that either did or did not receive the sample treatments (Figure 6). In the presence of appropriate cytokines, MDSCs differentiate into mature myeloid cells [35]. Such differentiation is blocked in the presence of tumor-cell-conditioned media or in tumor-bearing hosts [35]. Overall, our study might indicate that tumor-derived factors that induced expansion of MDSCs were unaffected by the sterol fraction of *P. dentata*. This would suggest other mechanisms of action on MDSC resulting in the inhibition of breast cancer tumorgenesis. 

The T cell immunosuppressive functions of MDSCs act through the release of short-lived soluble mediators such as ROS [6, 10] and arginase activity [12, 13]. Arginase catalyzes the conversion of L-arginine to urea and L-ornithine [8]. Recent data showed that there is a close correlation between the availability of L-arginine and the regulation of T cell proliferation [12, 13]. The high expression of arginase activity by MDSCs favors their direct inhibition on T-cell function [8, 11]. Serafini et al. [36] found that a phosphodiesterase-5 inhibitor, sildenafil, could downregulate the expression of arginase by MDSCs and, accordingly, inhibit the suppressive function of MDSCs in growing tumors. In this study the sterol fraction of *P. dentata* significantly decreased the ROS production (Figure 7(a)) and arginase activity (Figure 8) of MDSCs in 4T1 tumor-cell-engrafted mice *in vivo*. Accordingly, this fraction could down-regulate suppressive activity of MDSCs and decrease the tumor size (Figure 5). In addition, L-arginine is also used by nitric oxide synthase for nitric oxide (NO) generation [3]. Boucher et al. [37] showed that a low concentration of L-arginine resulted in low NO generation and high production of superoxide ion (^•^O_2_^−^). In this study, the high arginase activity in tumor-derived MDSCs in control mice (Figure 8) might have lowered the level of L-arginine, which resulted in increasing of ^•^O_2_^−^, one of ROS (Figures 6(a) and 6(b)) and undetectable NO (data not shown). *In vitro* the addition of an arginase inhibitor, Nor-NOHA, in MDSCs might cause the high level of L-arginine that can be used for NO production and, finally, significantly decreased ROS production (Figure 7(b)). 

## 5. Conclusion

This study was the first to screen and isolate the *P. dentata* fraction (F2 fraction) and to examine its potent anti-cancer activity. These anti-cancer effects probably result from the presence of *β*-sitosterol and campesterol. This sterol containing fraction reduced tumorgenesis and increased the survival rate of 4T1-engrafted mice; we suggest 2 likely mechanisms for this effect. First, the sample might cause the apoptosis of 4T1 cells. The other possible mechanism is that the sample might down-regulate the suppressive activity of MDSCs by affecting their ROS accumulation and arginase activity. This inhibition would be consistent with the use of *Porphyra dentata *as a folk medicine to treat inflammatory disorders and also for breast cancer.
